# Evaluation of patient safety culture among community pharmacists in Ethiopia: A cross-sectional study

**DOI:** 10.1371/journal.pone.0237338

**Published:** 2020-08-14

**Authors:** Malede Berihun Yismaw, Zelalem Tilahun Tesfaye, Haftom Gebregergs Hailu, Henok Getachew Tegegn, Eyob Alemayehu Gebreyohannes

**Affiliations:** 1 Department of Pharmacology and Clinical Pharmacy, School of Pharmacy, College of Health Sciences, Addis Ababa University (AAU), Addis Ababa, Ethiopia; 2 Department of Pharmacology and Toxicology, School of pharmacy, College of Health Sciences, Mekelle University, Mekelle, Ethiopia; 3 Department of Clinical Pharmacy, College of Medicine and Health Sciences, University of Gondar, Gondar, Ethiopia; Independent Researcher, UNITED KINGDOM

## Abstract

**Objective:**

The study was aimed to explore patient safety culture of community pharmacists working in Dessie and Gondar towns, Northern Ethiopia.

**Methods:**

A cross-sectional study was conducted from 1^st^ to 31^st^ March 2018. In this cross-sectional survey, the Pharmacy Survey on Patient Safety Culture (PSOPSC), developed by the Agency for Healthcare Research and Quality (AHRQ), was used to collect data. PSOPSC is a self-administered questionnaire. The questionnaire was distributed among staffs who work in community pharmacies of Dessie and Gondar towns. All staffs available on data collection period in the pharmacy were included. The Statistical Package for Social Science (SPSS) software version 25 was used to enter and analyze the data.

**Results:**

A total of 120 participants were approached and completed the questionnaire. Results from the study showed that high positive response rate was demonstrated in the domains of “Teamwork” (90.2%) followed by physical space and environment (83.1%). On the other hand, the result also identified that there is an enormous problem related to mistake communication (44.8%) and work pressure (45%). In addition, significant difference of percent positive responses were obtained across towns and staff working hours.

**Conclusions:**

The patient safety culture of community pharmacists is appreciable especially with respect to their teamwork. Besides, urgent attention should be given to areas of weakness, mainly in the domain of “mistake communication” and “staffing and work pressure”.

## Introduction

Patient safety has become a major global concern. It is described as "the freedom from accidental injuries during medical care; activities to avoid, prevent, or correct adverse outcomes which may result from the delivery of healthcare” [1]. Globally, there is an initiative and commitment to curb severe, preventable harm due to medications by 50% within five years through preventing errors or unsafe practices in healthcare systems [2]. Many injuries and deaths due to these medical errors, triggered the development of evidence-based strategies for better patient safety [3].

The safety culture in an organization can be defined as “the product of individual and group values, attitudes, perceptions, competencies, and patterns of behaviour that determine the commitment to, and the style and proficiency of, an organization’s health and safety management” [4]. The provision of safe quality care is no accident. Instead, organizations should integrate safety into their system to identify risk proactively, thereby lessening and, eventually, prevention of harm [3]. For an organization to have a better safety culture, there should exist effective communications, common understanding of the necessity of safety, and competencies for preventive measures [4]. Poor safety culture, defects in care processes, and lack of motivation in leadership could weaken delivery of quality and safe healthcare [5].

Community pharmacy is a necessary but overlooked part of the healthcare system as it could contribute to better patient safety through optimizing medication safety among patients and the community at large. Moreover, community pharmacies are continually striving to promote patient safety and quality. As a result, there is growing consideration and awareness of the importance of promoting patient safety culture. However, little is known regarding patient safety culture in community pharmacy [6, 7]. Most patient safety studies employed tools that are used to assess patient safety in hospitals [8–10].

Over the last two decades, Ethiopia has effectively implemented its strategy of expanding and rehabilitating primary health care facilities, led by mixture of public, private and nongovernmental healthcare sectors. Though promising outcomes are observed, the country is quiet challenged with triple burden of diseases consisting of non-communicable diseases, communicable diseases, and injuries which warrants strong commitment of healthcare sectors including community pharmacies [11].

In Ethiopia, there is limited evidence concerning patient safety culture in the healthcare system in general. Few studies on patient safety were done in a hospital setting [12–15]. However, none of the studies were stressed on the community pharmacy setting, which is the most accessible to the public. To the best of our knowledge, this is the first study in Ethiopia done in the community pharmacy. Therefore, this study seeks to assess patient safety culture of community pharmacists working in two urban areas of Ethiopia.

## Materials and methods

### Study design and population

A cross-sectional study was conducted from 1^st^ to 31^st^ March 2018. This study was conducted at community pharmacies found in Dessie and Gondar towns, Northern Ethiopia. A convenient sampling was used to select these two towns and all community pharmacies which were opened in the data collection time were included. Staff members of community pharmacies that participated in the study include pharmacists, pharmacy technicians, supportive staffs and pharmacy students on apprenticeship. All staff members who volunteered to participate in the study were included. Accordingly, a total of 120 participants were considered for the final analysis.

### Data collection

Data was collected using Pharmacy Survey on Patient Safety Culture (PSOPSC) tool, developed by the Agency for Healthcare Research and Quality (AHRQ) [16]. The questionnaire includes 36 items that measure 11 dimensions of patient safety culture as well as questions related to documentation of mistakes, respondents’ demographics and overall rating of the pharmacy. The demographic section was modified to fit the study population. The items for the parts were measured using the 5-point Likert response scale of agreement (Strongly Disagree to Strongly Agree) or frequency (Never to Always). The PSOPSC questionnaire was developed in English. It was translated into Amharic and then retranslated to English to maintain consistency. Data was collected by physically delivering the questionnaire to the community pharmacies where the study participants work.

### Data entry and analysis

The data collected was assessed for completeness; entered into and analyzed by Statistical Package for Social Sciences (SPSS) software version 25. Descriptive statistics including frequency, percentage, mean, standard deviation (SD) were used to represent the data. Pearson's chi-squared (χ^2^) and Fisher's exact tests were used to determine difference in patient safety culture across groups. A p value < 0.05 was taken as indicator of statistical significance.

### Ethics approval

Ethical clearance was obtained from Ethical Review Board of School of Pharmacy, University of Gondar. All participants provided informed written consent to participate in the study. Moreover, privacy of personal information and confidentiality of data was ensured throughout the study.

## Results

Out of 120 study participants who completed the survey, more than half (54.2%) were male, 57.5% worked in the town of Dessie, with the majority (74.2%) working in community pharmacies. The age of the study subjects ranged from 19 years to 62 years with a mean value of 29.47 ± 9.05 years. The background information of the subjects is summarized in Table 1.

**Table 1:** Demographic characteristics of respondents.

Variables	Frequency (%)
Sex	
Male	65 (54.2)
Female	55 (45.8)
Age (Mean ± SD)	29.47 ± 9.05
19–25	53 (44.2%)
26–35	44 (36.7)
36–45	15 (12.5)
> 45	8 (6.7)
Town	
Gondar	51 (42.5)
Dessie	69 (57.5)
Type of the facility	
Drug store	31 (25.8)
Community pharmacy	89 (74.2)
Work experience in the facility	
< 6 months	50 (41.7)
6 months to 1 year	11 (9.2)
2 years to 3 years	34 (28.3)
4 years to 6 years	13 (10.8)
7years to 12 years	11 (9.2)
13 years and above	1 (0.8)
Weekly working hours	
1–16 hours	23 (19.2)
17–31 hours	10 (8.3)
32–40 hours	28 (23.3)
> 40 hours	59 (49.2)

Regarding the statements related to physical space and environment, about 87.3% agreed that the pharmacy is free of clutter followed by good organization of the pharmacy (81.3%). On the subject of teamwork, more than 95% of the participants treat each other with respect and 88.2% reported that the staff work together as an effective team. However, about 32.4% of participants reported that technicians do not receive the training they need to do their jobs and 20.2% reported that staff did not get enough training from their respective setting. On the domains of communication openness, three quarter (75.6%) of the staff feel comfortable to ask questions when they are unsure about something whereas about a fifth (18.4%) of the staff reported that there is a barrier for staff members to speak up to their supervisor/ manager about patient safety concerns in their working environment. With respect to patient counselling, a significant number (86.6%) of participants mentioned that pharmacists tell patients important information about their new prescriptions. Meanwhile, interruptions/distractions in their pharmacy (from phone calls, faxes, customers, etc.) make it difficult for staff to work accurately according to 29.3% of participants. In addition, 61.5% of the study subjected stated that there was poor communication on the status of problematic prescriptions across shifts. According to the study participants, a large proportion (79.6%) of pharmacies help the staff to learn from their mistakes rather than punishing them whereas half of the participants reported that staffs feel like their mistakes are held against them. When the same mistake keeps happening, 76.8% of them changed the way how they did things. Mistakes lead to a positive change in 66.0% of respondents (Table 2).

**Table 2:** Response rate of individual items and dimensions positivity across community pharmacies.

Individual items and dimensions	Positive response N (%)	Negative response N (%)	Neutral response N (%)
**1. Physical space and environment (PRR = 83.1)**			
A1. This pharmacy is well organized (n = 107)	87 (81.3)	8 (7.5)	12 11.2)
A5. This pharmacy is free of clutter (n = 110)	96 (87.3)	5 (4.5)	9 (8.2)
A7. The physical layout of this pharmacy supports good workflow (n = 109)	88 (80.7)	5 (4.6)	16 (14.7)
**2. Teamwork (PRR = 90.2)**			
A2. Staff treat each other with respect (n = 110)	105 (95.5)	2 (1.8)	3 (2.7)
A4. Staff in this pharmacy clearly understand their roles and responsibilities (n = 108)	94 (87.0)	5 (4.6)	9 (8.3)
A9. Staff work together as an effective team (n = 110)	97 (88.2)	7 (6.4)	6 (5.5)
**3. Staff training and skills (PRR = 70.5)**			
A3. Technicians in this pharmacy receive the training they need to do their jobs (n = 105)	49 (46.7)	34 (32.4)	21.0)
A6. Staff in this pharmacy have the skills they need to do their jobs well (n = 110)	97 (88.2)	5 (4.5)	8 (7.3)
A8. Staff who are new to this pharmacy receive adequate orientation (n = 105)	90 (85.7)	7 (6.7)	8 (7.6)
A10. Staff get enough training from this pharmacy (n = 104)	64 (61.5)	21 (20.2)	19 (18.3)
**4. Communication openness (PRR = 67.3)**			
B1. Staff ideas and suggestions are valued in this pharmacy (n = 114)	67 (58.8)	15 (13.2)	(28.1)
B5. Staff feel comfortable asking questions when they are unsure about something (n = 119)	90 (75.6)	9 (7.6)	20 (16.8)
B10. It is easy for staff to speak up to their supervisor/ manager about patient safety concerns in this pharmacy (n = 114)	77 (67.5)	21 (18.4)	16 (14.0)
**5. Patient counselling (PRR = 80.7)**			
B2. We encourage patients to talk to pharmacists about their medications (n = 119)	97 (81.5)	10 (8.4)	12 (10.1)
B7. Our pharmacists spend enough time talking to patients about how to use their medications (n = 117)	87 (74.4)	13 (11.1)	17 (14.5)
B11. Our pharmacists tell patients important information about their new prescriptions (n = 119)	103 (86.6)	3 (2.5)	13 (10.9)
**6. Staffing and work pressure (PRR = 45)**			
B3. Staff take adequate breaks during their shifts (n = 116)	55 (47.4)	29 (25.0)	32 27.6)
B9. We feel rushed when processing prescriptions (n = 118) *	38 (32.2)	32 (27.1)	48 (40.7)
B12. We have enough staff to handle the workload (n = 117)	78 (66.7)	19 (16.2)	20 17.1)
B16. Interruptions/distractions in this pharmacy (from phone calls, faxes, customers, etc.) make it difficult for staff to work accurately (n = 116) *	39 (33.6)	34 (29.3)	43 37.1)
**7. Communication across shifts (PRR = 52.8)**			
B4. We have clear expectations about exchanging important prescription information across shifts (n = 110)	50 (45.5)	35 (31.8)	25 (22.7)
B6. We have standard procedures for communicating prescription information across shifts (n = 109)	56 (51.4)	27 (24.8)	26 (23.9)
B14. The status of problematic prescriptions is well communicated across shifts (n = 117)	72 (61.5)	16 (13.7)	29 (24.8)
**8. Mistakes communication (PRR = 44.8)**			
B8. Staff in this pharmacy discuss mistakes (n = 116)	0 (0.0)	48 (41.4)	68 (58.6)
B13. When patient safety issues occur in this pharmacy, staff discuss them (n = 112)	68 (60.7)	18 (16.1)	26 (23.2)
B15. In this pharmacy, we talk about ways to prevent mistakes from happening again (n = 114)	84 (73.7)	13 (11.4)	17 (14.9)
**9. Response to mistakes (PRR = 66.4)**			
C1. Staff are treated fairly when they make mistakes (N = 112)	82 (73.2)	15 (13.4)	15 (13.4)
C4. This pharmacy helps staff learn from their mistakes rather than punishing them (N = 113)	90 (79.6)	8 (7.1)	15 (13.3)
C7. We look at staff actions and the way we do things to understand why mistakes happen in this pharmacy (N = 113)	71 (62.8)	22 (19.5)	20 (17.7)
C8. Staff feel like their mistakes are held against them (N = 106) *	53 (50)	33 (31.1)	20 (18.9)
**10. Organizational learning-continuous improvement (PRR = 71.2)**			
C2. When a mistake happens, we try to figure out what problems in the work process led to the mistake (N = 117)	83 (70.9)	12 (10.3)	22 (18.8)
C5. When the same mistake keeps happening, we change the way we do things (112)	86 (76.8)	14 (12.5)	12 (10.7)
C10. Mistakes have led to positive changes in this pharmacy (103)	68 (66.0)	23 (22.3)	12 (11.7)
**11. Overall perception of patient safety (PRR = 76.7)**			
C3. This pharmacy places more emphasis on sales than on patient safety (N = 117) *	89 (76.1)	0 (0)	28 (23.9)
C6. This pharmacy is good at preventing mistakes (N = 111)	85 (76.6)	14 (12.6)	12 (10.8)
C9. The way we do things in this pharmacy reflects a strong focus on patient safety (N = 112)	86 (76.8)	12 (10.7)	14 (12.5)

PRR: Positive response rate;

*Negatively worded items are reversed coded

The result of the survey also showed that the majority of participants did not carry out any documentation of mistakes. Similarly, there is no documentation in 59% of cases when a mistake that could have harmed the patient is corrected before the medication leaves the pharmacy (Table 3).

**Table 3:** Response rate of individual items on the domain of “Documentation of mistakes”.

Documentation of mistakes	Always	Never	Sometimes
D1. When a mistake reaches the patient and could cause harm but does not, how often is it documented? (N = 99)	8 (8.1)	75 (75.8)	16 (16.1)
D2. When a mistake reaches the patient but has no potential to harm the patient, how often is it documented? (99)	10 (10.1)	74 (74.7)	15 (15.2)
D3. When a mistake that could have harmed the patient is corrected BEFORE the medication leaves the pharmacy, how often is it documented? (N = 100)	18 (18.0)	59 (59.0)	23 (23.0)

The overall rating of the pharmacy on patient safety was excellent in 33% of study subjects followed by very good (30.8%), good (25.1%), fair (7.5%) and poor (3.3%).

The overall percent positive responses on 11 dimensions ranges from 45%-90.2% with average percent positive response of 68.1%. The results of positive responses’ percentage on patient safety culture composites was higher (90.2%) with respect to teamwork followed by physical space and environment (83.1%). Conversely, a much lower positive response were found in statements assessing the “mistake communication” and “staffing and work pressure” (44.8%, 45% respectively) as depicted in Table 2.

Analysis of the data showed a significant difference in 16 dimensions between Gondar and Dessie town. The positive response rate of all the 16 items was higher in Dessie town participants: *(1) This pharmacy is free of clutter*, *(2) Staff work together as an effective team*, *(3) Staff who are new to this pharmacy receive adequate orientation*, *(4) Staff ideas and suggestions are valued in this pharmacy*, *(5) Our pharmacists spend enough time talking to patients about how to use their medications*, *(6) Our pharmacists tell patients important information about their new prescriptions*, *(7) We have clear expectations about exchanging important prescription information across shifts*, *(8) In this pharmacy*, *we talk about ways to prevent mistakes from happening again*, *(9) Staff are treated fairly when they make mistakes*, *(10) This pharmacy helps staff learn from their mistakes rather than punishing them*, *(11) When a mistake happens*, *we try to figure out what problems in the work process led to the mistake*, *(12) When the same mistake keeps happening*, *we change the way we do things*, *(13) Mistakes have led to positive changes in this pharmacy*, *(14) This pharmacy places more emphasis on sales than on patient safety*, *(15) This pharmacy is good at preventing mistakes and (16) The way we do things in this pharmacy reflects a strong focus on patient safety* (p<0.05).

The provision of patient safety culture was also closely related to the participants’ length of working hours per week. Our analysis showed that there was a significant difference in 14 dimensions as illustrated in Table 4.

**Table 4:** The comparison of attitudes of participants in the two towns and their length of working hours on patient safety culture.

Items	Town		P value	Working hours per week				P value
	Gondar	Dessie		1–16	17–31	32–40	>40	
	PRR	PRR		PRR	PRR	PRR	PRR	
A1. This pharmacy is well organized (n = 107)	75.0	85.1	0.196^a^	62.5	88.9	84.0	84.2	0.261^b^
A5. This pharmacy is free of clutter (n = 110)	68.3	98.6	**<0.001**^**a**^	56.3	100.0	84.6	94.9	**0.001**^**b**^
A7. The physical layout of this pharmacy supports good workflow (n = 109)	73.2	85.3	0.120^a^	68.8	100.0	68.0	86.4	0.056^b^
A2. Staff treat each other with respect (n = 110)	92.7	97.1	0.358^b^	87.5	100.0	96.2	96.6	0.460^b^
A4. Staff in this pharmacy clearly understand their roles and responsibilities (n = 108)	80.0	91.2	0.095^a^	87.5	100.0	88.0	84.5	0.815^b^
A9. Staff work together as an effective team (n = 110)	75.6	95.7	**0.004**^**b**^	93.8	88.9	84.6	88.1	0.903^b^
A3. Technicians in this pharmacy receive the training they need to do their jobs (n = 105)	50.0	44.6	0.591^a^	78.6	50.0	20.0	50.0	**0.003**^**b**^
A6. Staff in this pharmacy have the skills they need to do their jobs well (n = 110)	80.5	92.8	0.070^b^	87.5	100.0	84.6	88.1	0.822^b^
A8. Staff who are new to this pharmacy receive adequate orientation (n = 105)	67.6	95.6	**<0.001**^**a**^	76.9	100.0	76.0	89.7	0.155^b^
A10. Staff get enough training from this pharmacy (n = 104)	60.5	62.1	0.872^a^	76.9	66.7	50.0	62.1	0.440^b^
B1. Staff ideas and suggestions are valued in this pharmacy (n = 114)	44.7	68.7	**0.010**^**a**^	10.0	80.0	60.7	71.4	**<0.001**^**b**^
B5. Staff feel comfortable asking questions when they are unsure about something (n = 119)	68.0	81.2	0.099^a^	56.5	80.0	67.9	86.2	**0.024**^**b**^
B10. It is easy for staff to speak up to their supervisor/ manager about patient safety concerns in this pharmacy (n = 114)	59.6	73.1	0.128^a^	33.3	90.0	74.1	73.2	**0.003**^**b**^
B2. We encourage patients to talk to pharmacists about their medications (n = 119)	74.0	87.0	0.072^a^	60.9	90.0	75.0	91.4	**0.009**^**b**^
B7. Our pharmacists spend enough time talking to patients about how to use their medications (n = 117)	59.2	85.3	**0.001**^**a**^	39.1	80.0	77.8	86.0	**<0.001**^**b**^
B11. Our pharmacists tell patients important information about their new prescriptions (n = 119)	74.0	95.7	**0.001**^**a**^	63.6	90.0	89.3	93.2	**0.010**^**b**^
B3. Staff take adequate breaks during their shifts (n = 116)	44.9	49.3	0.643^a^	54.5	60.0	38.5	46.6	0.585^b^
B9. We feel rushed when processing prescriptions (n = 118) *	39.2	26.9	0.155^a^	26.1	50.0	44.4	25.9	0.185^b^
B12. We have enough staff to handle the workload (n = 117)	60.0	71.6	0.186^a^	43.5	60.0	65.4	77.6	**0.030**^**b**^
B16. Interruptions/distractions in this pharmacy (from phone calls, faxes, customers, etc.) make it difficult for staff to work accurately (n = 116) *	32.0	34.8	0.748^a^	30.4	60.0	40.7	26.8	0.174^b^
B4. We have clear expectations about exchanging important prescription information across shifts (n = 110)	27.9	56.7	**0.003**^**a**^	30.0	60.0	53.8	44.4	0.326^b^
B6. We have standard procedures for communicating prescription information across shifts (n = 109)	43.2	56.9	0.159^a^	35.0	70.0	64.0	48.1	0.156^b^
B14. The status of problematic prescriptions is well communicated across shifts (n = 117)	53.1	67.6	0.110^a^	31.8	80.0	66.7	67.2	**0.015**^**b**^
B8. Staff in this pharmacy discuss mistakes (n = 116)	0.0	0.0	**	100.0	100.0	100.0	100.0	**
B13. When patient safety issues occur in this pharmacy, staff discuss them (n = 112)	55.6	64.2	0.360^a^	47.4	80.0	60.0	62.1	0.409^b^
B15. In this pharmacy, we talk about ways to prevent mistakes from happening again (n = 114)	62.2	81.2	**0.025**^**a**^	63.2	70.0	81.5	74.1	0.535^b^
C1. Staff are treated fairly when they make mistakes (N = 112)	58.3	84.4	**0.002**^**a**^	55.0	80.0	68.0	80.7	0.141^b^
C4. This pharmacy helps staff learn from their mistakes rather than punishing them (N = 113)	56.5	95.5	**<0.001**^**a**^	60.0	80.0	69.2	91.2	**0.007**^**b**^
C7. We look at staff actions and the way we do things to understand why mistakes happen in this pharmacy (N = 113)	55.6	67.6	0.193^a^	50.0	80.0	60.0	65.5	0.427^b^
C8. Staff feel like their mistakes are held against them (N = 106) *	60.5	42.9	0.075^a^	22.2	60.0	29.2	66.7	**0.001**^**a**^
C2. When a mistake happens, we try to figure out what problems in the work process led to the mistake (N = 117)	49.0	86.8	**<0.001**^**a**^	45.5	70.0	76.9	78.0	**0.040**^**b**^
C5. When the same mistake keeps happening, we change the way we do things (112)	60.0	88.1	**0.001**^**a**^	66.7	80.0	68.0	83.9	0.245^b^
C10. Mistakes have led to positive changes in this pharmacy (103)	50.0	77.0	**0.004**^**a**^	61.1	80.0	68.2	64.2	0.809^b^
C3. This pharmacy places more emphasis on sales than on patient safety (N = 117) *	51.0	95.5	**<0.001**^**a**^	26.1	77.8	77.8	94.8	**<0.001**^**b**^
C6. This pharmacy is good at preventing mistakes (N = 111)	58.7	89.2	**<0.001**^**a**^	63.2	70.0	76.0	82.5	0.331^b^
C9. The way we do things in this pharmacy reflects a strong focus on patient safety (N = 112)	58.7	89.4	**<0.001**^**a**^	73.7	80.0	72.0	79.3	0.881^b^
D1. When a mistake reaches the patient and could cause harm but does not, how often is it documented? (N = 99)	6.7	9.3	0.725^b^	5.6	0.0	16.0	6.3	0.478^b^
D2. When a mistake reaches the patient but has no potential to harm the patient, how often is it documented? (99)	6.7	13.0	0.340^b^	10.5	12.5	16.0	6.4	0.518^b^
D3. When a mistake that could have harmed the patient is corrected BEFORE the medication leaves the pharmacy, how often is it documented? (N = 100)	15.6	20.0	0.565^a^	15.8	12.5	32.0	12.5	0.222^b^

P-values were generated using ^a^Pearson chi-square test and ^b^Fisher’s exact test; Significant numbers from the statistical tests were presented in bold;

* Negatively worded items were reversed coded;

**No statistics are computed because Staff in this pharmacy discuss mistakes is a constant; PRR- Positive response rate

## Discussion

This study is the first of its kind done in Ethiopia to assess patient safety culture of community pharmacists in their practice setting. Assessing the patent safety culture of healthcare professionals is an indeed area to build trust between healthcare personals and clients and eventually improves patients’ quality of care.

In the present study, the positive response rate (PRR) was calculated for 11 patient safety culture composites, and the result showed that, except mistake communication and work pressure, all composites received more than 50% scores. The results have also illustrated remarkable variations (45%-90.2%) in the PRR across the domains with mean overall PRR of 68.1%. The mean overall PRR was comparable with study findings reported from Sweden [17], Taiwan [18], United Arab Emirates [19] and Kuwait [6] (61.2%, 65%, 74.7%, and 83.8%, respectively).

The highest PRR on patient safety culture composites was obtained on questions assessing teamwork (90.2%). In accordance with our study, teamwork received a higher PRR (76–94%) in other studies conducted in China [20], Turkey [21], Sweden [17] and Taiwan [18]. The reason for this high score might be due to the fact that pharmacy professionals’ work in settings that have working stations near each other. For instance, a pharmacist may have a pharmacy technician, another fellow pharmacist, and a cashier who all work within a distance of few feet. Such proximity at the work room may facilitate teamwork.

Effective teamwork is an indispensable part for provision of quality care for patients. Pharmacy professionals should contribute to the training and development of the team, education, and must delegate tasks only to people who are competent and appropriately trained. Training and development for pharmacy teams should be given to acquire knowledge and skills needed to meet the new challenges and opportunities they face in their working area [22].

Pharmacy leaders who can create psychologically safe environments and engage their team members with a shared purpose can use teamwork to improve patient outcomes and employee welfare [23].

Statements related to physical space and environment (83.1%) received the second highest PRR among the 11 patient safety culture composites finding. This percentage was slightly lower than a study reported in Kuwait (88.2%) [6] but higher than study done in Wisconsin (73%) [24].

The availability of physical space and good environment at the working area influenced the dispensing process and patient safety. Space for physical movement, storage and prescription processing facilitated the flexibility of the dispensing process. In pharmacies that had limited space, dispensing appeared chaotic as staff bumped into each other whilst going about tasks, or could not queue items on counter tops for processing and sometimes piled items into basket; the baskets were then piled on top of each other. Medicines were sometimes stored in cardboard boxes on the floor where shelves were full.

As community pharmacy practice is increasingly becoming more involved in advanced medication and disease state management services with unique privacy requirements, pharmacies’ layouts and systems to address privacy challenges require a proactive approach [25]. Thus, an adequate physical environment in the working area helps the pharmacist to provide better clinical care.

The lowest PRR were obtained on questions assessed mistake communication (44.8%) and work pressure (45%). Similarly, staffing, work pressure, and pace received the lowest PRR (37%) of all dimensions is a study conducted by Aboneh et al. Their finding also showed that communication about mistakes received the second least (71%) PRR score [24].

However, a higher PRR of communication about Mistakes (81.8%) was reported in a study conducted Alsaleh et al., while statements assessed staffing, work pressure, and pace (49.7%) exhibited a similar score to our findings [6].

Despite being cornerstones of therapy in healthcare, medications remained to be common sources of mistakes and harms, which in most instances are preventable.

As human beings, we all are prone to make mistakes and drift into unsafe behavioural choices while failing to appreciate the risk, regardless of how well the system is designed or how careful and vigilant we intend to be. There should be communication on errors occurred to prevent further mistake occurrence [26].

According to Institute of Medicine “The biggest challenge to moving toward a safer health system is changing the culture from one of blaming individuals for errors to one in which errors are treated not as personal failures, but as opportunities to improve the system and prevent harm” [27].

Mistakes can be minimized using automated technologies. For instance, development of automated dispensing systems (ADS) have shown significant effect on minimizing dispensing errors [28].

Working in more stressful working environments for long hours, community pharmacists are predisposed to increased job-related stress and decreased job satisfaction, which subsequently impacts the amount and quality of information and service they provide to patients [28–30]. Consequently, the environment should be made safer to deliver quality service.

The overall rating of the pharmacy on patient safety was rated at least “good” in most (89.2%) of the participants, implying a strong sense of appreciation of their pharmacies’ value towards upholding patient safety culture.

The result was similar to a study conducted in Wisconsin which reported overall rating was very high (Good 21%, Very good 44% and Excellent 26%) [24].

A study done in Kuwait also reported that the overall grade of patient safety rating given by the pharmacists to their pharmacy was very high (Good 25.3%, Very good 50.2% and Excellent 20.6%) [6].

In the current study, there was a significant difference in 16 dimensions across the two (Gondar and Dessie) towns. As a result, participants from community pharmacies in Dessie town showed higher rate of positive response in all of the 16 dimensions, more than half of which were associated with prevention and correction of mistakes. The difference in the positive response rates across the towns may be attributed to several reasons including the fact that Dessie town has the largest number of community pharmacies in the region while having smaller population as compared to Gondar town, hence making customer overload, working pressure, and associated errors less likely than community pharmacies in Gondar town. Moreover, it can be stipulated that the abundance of community pharmacies in Dessie encourages competition with one another leading to better patient safety profiles in those institutions. Similar argument was forwarded by a study from Kuwait, which reported lowest safety scores in states with lower community pharmacy to population ratios [6]. This implies the need for planning and implementing measures to match the number of community pharmacies to the population size in a given town or city.

Significant difference in the rate of positive response was also noted over 14 dimensions across participants with different weekly working hours. Accordingly, participants working more than 40 hours a week scored higher positive responses to dimensions related to communication and workplace. This may be because the staff members who spend longer in the pharmacy premises get more opportunities to develop better communication with co-workers and patients.

Though the study is new in addressing issues related to handling of patients’ in Ethiopian community pharmacies, it has its own limitations. Using a self-administered data collection tool leads to response bias due to the fear of the impact of negative responses on the pharmacies’ reputation and thus pharmacists’ job security. Besides, due to lack of similar studies done in other African nations, we were unable to compare our findings with these nations, which have comparable socio-economic status and public health burden. Findings might also be limited by selection bias as pharmacies were selected on a convenience basis.

## Conclusion

The patient safety culture of community pharmacists is appreciable especially with respect to their teamwork. But there is a considerable lesser positive response on statements related to “mistake communication” and “staffing and work pressure”. So, urgent attention should be given to these areas of weakness. In addition, there is significant difference in maintaining patient safety culture across towns and length of working hours per week.

## Supporting information

S1 Data (DOC)
