# Supplementary material for: Evaluation of patient safety culture among community pharmacists in Ethiopia: A cross-sectional study
Source: PLoS One. 2020 Aug 14;15(8):e0237338. doi: 10.1371/journal.pone.0237338 (PMC7428191; doi:10.1371/journal.pone.0237338)
Supplement: S1 Data — (DOC) [file pone.0237338.s001.doc]

**Data Abstraction Tool**

**Community Pharmacy Survey on Patient Safety**

This survey asks for your opinions about patient safety in this community pharmacy and takes about 15 minutes to complete. Answer only about the pharmacy location/store where you received this survey.


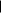


- **Staff** means **EVERYONE who works in this community pharmacy,** including pharmacists, pharmacy technicians, pharmacy clerks, etc.
- **Patient safety** is the prevention of patient harm resulting from the processes of health care delivery. In the pharmacy setting, it means that:
  - The right patient receives the right medication in the right dose at the right time by the right route, and
  - The patient or caregiver understands the purpose and proper use of the medication.
- A **mistake** is any type of medication error, mistake, incident, or quality-related event, regardless of whether or not it reaches the patient or results in patient harm. Mistakes may be related to, or include:
  - Prescribing, transcribing, dispensing, administering, monitoring (use of medication), unsafe conditions or procedures in the pharmacy, etc.
- If a question does not apply to you or you don’t know the answer, please answer “Does Not Apply or Don’t Know.”

**
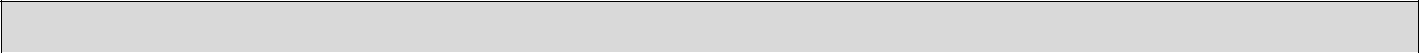
**

**SECTION A: Working in This Pharmacy**

**
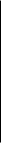
**

| **How much do you agree or disagree with the** | |  |  | **Neither** |  |  | **Does Not** |
| --- | --- | --- | --- | --- | --- | --- | --- |
|  |  |  |  | **Agree** |  |  | **Apply or** |
| **following statements?** *Remember, “staff”* *means* | | **Strongly** |  | **nor** |  | **Strongly** | **Don’t** |
| *everyone working in this pharmacy.* | | **Disagree** | **Disagree** | **Disagree** | **Agree** | **Agree** | **Know** |
|  |  | ⯆ | ⯆ | **⯆** | ⯆ | ⯆ | ⯆ |
|  |  |  |  |  |  |  |  |
| 1. | This pharmacy is well organized ................................ | 1 | 2 | 3 | 4 | 5 | 9 |
| 2. | Staff treat each other with respect .............................. | 1 | 2 | 3 | 4 | 5 | 9 |
| 3. | Technicians in this pharmacy receive the training |  |  |  |  |  |  |
|  | they need to do their jobs ........................................... | 1 | 2 | 3 | 4 | 5 | 9 |
| 4. | Staff in this pharmacy clearly understand their |  |  |  |  |  |  |
|  | roles and responsibilities ............................................ | 1 | 2 | 3 | 4 | 5 | 9 |
| 5. | This pharmacy is free of clutter .................................. | 1 | 2 | 3 | 4 | 5 | 9 |
| 6. | Staff in this pharmacy have the skills they need |  |  |  |  |  |  |
|  | to do their jobs well ..................................................... | 1 | 2 | 3 | 4 | 5 | 9 |
| 7. | The physical layout of this pharmacy supports |  |  |  |  |  |  |
|  | good workflow ............................................................. | 1 | 2 | 3 | 4 | 5 | 9 |
| 8. | Staff who are new to this pharmacy receive |  |  |  |  |  |  |
|  | adequate orientation ................................................... | 1 | 2 | 3 | 4 | 5 | 9 |
| 9. | Staff work together as an effective team .................... | 1 | 2 | 3 | 4 | 5 | 9 |
| 10. | Staff get enough training from this pharmacy............. | 1 | 2 | 3 | 4 | 5 | 9 |
|  |  |  |  |  |  |  |  |


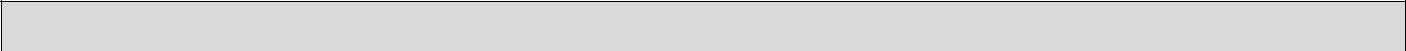


**SECTION B: Communication and Work Pace**

|  |  |  |  |  |  |  |  |  |  | **Does Not** |
| --- | --- | --- | --- | --- | --- | --- | --- | --- | --- | --- |
|  |  |  |  |  |  |  |  |  |  | **Apply or** |
| **How often do the following statements apply to** | | | | |  |  | **Some-** | **Most of** |  | **Don’t** |
|  |  |  |  |  | **Never** | **Rarely** | **times** | **the time** | **Always** | **Know** |
| **this pharmacy?** | | | | |  |  |  |  |  |  |
|  |  |  |  |  | ⯆ | ⯆ | **⯆** | ⯆ | ⯆ | ⯆ |
|  |  |  |  |  |  |  |  |  |  |  |
| 1. | Staff ideas and suggestions are valued in this | | | |  |  |  |  |  |  |
|  |  |  |  |  |  |  |  |  |  |  |
|  | pharmacy .................................................................... | | | | 1 | 2 | 3 | 4 | 5 | 9 |
| 2. | We encourage patients to talk to pharmacists | | | |  |  |  |  |  |  |
|  | about their medications ............................................ | | | | 1 | 2 | 3 | 4 | 5 | 9 |
| 3. | Staff take adequate breaks during their shifts.......... | | | | 1 | 2 | 3 | 4 | 5 | 9 |
| 4. | We have clear expectations about exchanging | | | |  |  |  |  |  |  |
|  | important prescription information across shifts....... | | | | 1 | 2 | 3 | 4 | 5 | 9 |
| 5. | Staff feel comfortable asking questions when | | | |  |  |  |  |  |  |
|  | they are unsure about something ............................ | | | | 1 | 2 | 3 | 4 | 5 | 9 |
| 6. | We have standard procedures for communicating | | | |  |  |  |  |  |  |
|  | prescription information across shifts ......................... | | | | 1 | 2 | 3 | 4 | 5 | 9 |
| 7. | Our pharmacists spend enough time talking to | | | |  |  |  |  |  |  |
|  | patients about how to use their medications ............ | | | | 1 | 2 | 3 | 4 | 5 | 9 |
| 8. | Staff in this pharmacy discuss mistakes .................... | | | | 1 | 2 | 3 | 4 | 5 | 9 |
| 9. | We feel rushed when processing prescriptions ....... | | | | 1 | 2 | 3 | 4 | 5 | 9 |


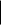


1. It is easy for staff to speak up to their supervisor/ manager about patient safety concerns in this

|  | pharmacy.................................................................. | 1 | 2 | 3 | 4 | 5 | 9 |
| --- | --- | --- | --- | --- | --- | --- | --- |
| 11. | Our pharmacists tell patients important information |  |  |  |  |  |  |
|  | about their new prescriptions ..................................... | 1 | 2 | 3 | 4 | 5 | 9 |
| 12. | We have enough staff to handle the workload ......... | 1 | 2 | 3 | 4 | 5 | 9 |
| 13. | When patient safety issues occur in this pharmacy, |  |  |  |  |  |  |
|  | staff discuss them ....................................................... | 1 | 2 | 3 | 4 | 5 | 9 |
| 14. | The status of problematic prescriptions is well |  |  |  |  |  |  |
|  | communicated across shifts ....................................... | 1 | 2 | 3 | 4 | 5 | 9 |
| 15. | In this pharmacy, we talk about ways to prevent |  |  |  |  |  |  |
|  | mistakes from happening again ................................. | 1 | 2 | 3 | 4 | 5 | 9 |
| 16. | Interruptions/distractions in this pharmacy |  |  |  |  |  |  |
|  | (from phone calls, faxes, customers, etc.) |  |  |  |  |  |  |
|  | make it difficult for staff to work accurately .............. | 1 | 2 | 3 | 4 | 5 | 9 |


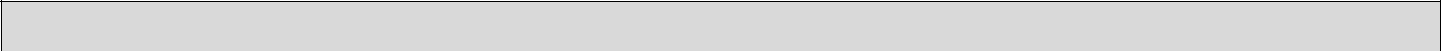
**SECTION C: Patient Safety and Response to Mistakes**

**
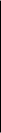
**

**Neither**

**Agree**

| **How much do you agree or disagree with the** | | | | **Strongly** |  | **nor** |  | **Strongly** | **Don’t** |
| --- | --- | --- | --- | --- | --- | --- | --- | --- | --- |
| **following statements?** | | | | **Disagree** | **Disagree** | **Disagree** | **Agree** | **Agree** | **Know** |
|  |  |  |  | ⯆ | ⯆ | **⯆** | ⯆ | ⯆ | ⯆ |
|  |  |  |  |  |  |  |  |  |  |
| 1. | Staff are treated fairly when they make mistakes ..... | | | 1 | 2 | 3 | 4 | 5 | 9 |
| 2. | When a mistake happens, we try to figure out what | | |  |  |  |  |  |  |
|  | problems in the work process led to the mistake ...... | | | 1 | 2 | 3 | 4 | 5 | 9 |
| 3. | This pharmacy places more emphasis on sales | | |  |  |  |  |  |  |
|  | than on patient safety ................................................ | | | 1 | 2 | 3 | 4 | 5 | 9 |
| 4. | This pharmacy helps staff learn from their mistakes | | |  |  |  |  |  |  |
|  | rather than punishing them ....................................... | | | 1 | 2 | 3 | 4 | 5 | 9 |
| 5. | When the same mistake keeps happening, we | | |  |  |  |  |  |  |
|  | change the way we do things .................................... | | | 1 | 2 | 3 | 4 | 5 | 9 |
| 6. | This pharmacy is good at preventing mistakes ......... | | | 1 | 2 | 3 | 4 | 5 | 9 |
| 7. | We look at staff actions and the way we do things | | |  |  |  |  |  |  |
|  |  |  |  |  |  |  |  |  |  |
|  | to understand why mistakes happen in this | | |  |  |  |  |  |  |
|  | pharmacy................................................................... | | | 1 | 2 | 3 | 4 | 5 | 9 |
| 8. | Staff feel like their mistakes are held against them .. | | | 1 | 2 | 3 | 4 | 5 | 9 |
| 9. | The way we do things in this pharmacy reflects | | |  |  |  |  |  |  |
|  | a strong focus on patient safety ................................ | | | 1 | 2 | 3 | 4 | 5 | 9 |
| 10. | Mistakes have led to positive changes in this | | |  |  |  |  |  |  |
|  | pharmacy................................................................... | | | 1 | 2 | 3 | 4 | 5 | 9 |

**Does Not**

**Apply or**

**
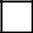

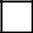

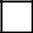

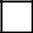

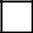

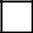
**

**SECTION D: Documenting Mistakes**

**In this pharmacy, how often are the following types of mistakes documented (in writing OR tracked electronically)?**

|  |  |  |  |  |  |  |  |  |  |  |  |  | |  |  |  |  | **Most of the** |  | **Does Not** |
| --- | --- | --- | --- | --- | --- | --- | --- | --- | --- | --- | --- | --- | --- | --- | --- | --- | --- | --- | --- | --- |
|  |  |  |  |  |  |  |  |  |  |  |  |  | |  | **Never** | **Rarely** | **Sometimes** | **time** | **Always** | **Apply or** |
|  |  |  |  |  |  |  |  |  |  |  |  |  | | **documented** | | **documented** | **documented** | **documented documented Don’t Know** | | |
|  |  |  |  |  |  |  |  |  |  |  |  |  | |  | ⯆ | ⯆ | **⯆** | ⯆ | ⯆ | ⯆ |
| 1. | | When a mistake reaches the patient | | | | | | | | | | | | |  |  |  |  |  |  |
|  |  | and could cause harm but does not, | | | | | | | | | | | | |  |  |  |  |  |  |
|  |  |  |  |  |  |  | |  |  |  |  |  | |  |  |  |  |  |  |  |
|  |  | how often is it documented? ................... | | | | | | | | | | | | | 1 | 2 | 3 | 4 | 5 | 9 |
| 2. | | When a mistake reaches the patient but | | | | | | | | | | | | |  |  |  |  |  |  |
|  |  | has no potential to harm the patient, | | | | | | | | | | | | |  |  |  |  |  |  |
|  |  |  |  |  |  |  | |  |  |  |  | |  | |  |  |  |  |  |  |
|  |  | how often is it documented? ................... | | | | | | | | | | | | | 1 | 2 | 3 | 4 | 5 | 9 |
| 3. | | When a mistake that could have | | | | | | | | | | | | |  |  |  |  |  |  |
|  |  |  | |  |  |  | |  | |  |  | |  | |  |  |  |  |  |  |
|  |  | harmed the patient is corrected | | | | | | | | | | | | |  |  |  |  |  |  |
|  |  |  | |  |  |  | | | |  | | |  | |  |  |  |  |  |  |
|  |  | BEFORE the medication leaves the | | | | | | | | | | | | |  |  |  |  |  |  |
|  |  |  | | | |  | | | | | | |  | |  |  |  |  |  |  |
|  |  | pharmacy, how often is it documented? | | | | | | | | | | | | | 1 | 2 | 3 | 4 | 5 | 9 |
|  |  |  |  |  |  |  |  |  |  |  |  |  | |  |  |  |  |  |  |  |

**SECTION E: Overall Rating**

1. **Think back on the survey topics and the definition of patient safety—dispensing the right medication accurately and making sure patients understand their medications and how to use them:**

**How do you rate this pharmacy on patient safety?**

| **Poor** | **Fair** | **Good** | **Very good** | **Excellent** |
| --- | --- | --- | --- | --- |
| **▼** | **▼** | **▼** | **▼** | **▼** |
| 1 | 2 | 3 | 4 | 5 |


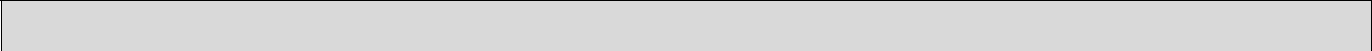


**SECTION F: Background Questions**

| Sex |
| --- |
| 🞏 Male |
| 🞏 Female |
| Age (in years)--------------- |
| 🞏 19 – 25 |
| 🞏 26 – 35 |
| 🞏 36 – 45 |
| 🞏 > 45 |
| Town |
| 🞏 Gondar |
| 🞏 Dessie |
| Type of the facility |
| 🞏 Drug store |
| 🞏Community pharmacy |

**1. How long have you worked in this pharmacy?**

**
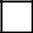
**

a. Less than 6 months


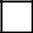


b. 6 months to less than 1 year c. 1 year to less than 3 years


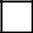


1. 3 years to less than 6 years
2. 6 years to less than 12 years
3. 12 years or more


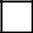

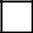

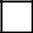


**2. Typically, how many hours per week do you work in this pharmacy?**

**
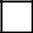

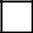
**

| a. | 1 to 16 hours per week | c. 32 to 40 hours per week |
| --- | --- | --- |
| b. | 17 to 31 hours per week | d. More than 40 hours per week |


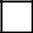

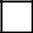


1. **What is your position in this pharmacy? *Check ONE category that best applies to your job.***

**
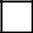
**

a. Pharmacist (including pharmacy manager, lead pharmacist, pharmacist-in-charge, staff pharmacist)


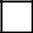


b. Pharmacy technician (including lead technician and staff technician)


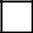


c. Pharmacy clerk or pharmacy cashier


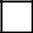


d. Pharmacy student intern/extern


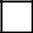


e. Other (Please write your job title): ___________________________________


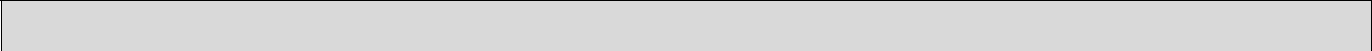


**SECTION G: Your Comments**

**Please feel free to write any comments about how things are done or could be done in your pharmacy that might affect patient safety.**

***THANK YOU FOR COMPLETING THIS SURVEY.***
